# Influence of domain walls thickness, density and alignment on Barkhausen noise emission in low alloyed steels

**DOI:** 10.1038/s41598-023-32792-1

**Published:** 2023-04-07

**Authors:** M. Neslušan, M. Pitoňák, P. Minárik, M. Tkáč, P. Kollár, O. Životský

**Affiliations:** 1 University of Žilina, Univerzitná 1, 010 26 Žilina, Slovakia; 2 Faculty of Mathematics and Physics, Charles University, Ke Karlovu 5, 121 16 Praha 2, Czech Republic; 3 Institute of Physics, Faculty of Science, P. J. Šafárik University in Košice, Park Angelium 9, 040 01 Kosice, Slovakia; 4 Faculty of Electrical Engineering and Computer Science, VŠB – Technical University of Ostrava, 17. Listopadu 2172/15, 708 00 Ostrava-Poruba, Czech Republic

**Keywords:** Engineering, Materials science, Physics

## Abstract

This study deals with the characterization of low alloyed steels of different yield strengths (varying in the range of 235–1100 MPa) via Barkhausen noise emission. The study investigates the potential of this technique to distinguish among the low alloyed steels and all significant aspects contributing to Barkhausen noise, such as the residual stress state, microstructure expressed in terms of dislocation density, grain size, prevailing phase, as well as associated aspects of the domain wall substructure (domain wall thickness, energy, their spacing and density in the matrix). Barkhausen noise in the rolling as well as transversal direction grows along with the yield strength (up to 500 MPa) and the corresponding grain refinement of ferrite. As soon as the martensite transformation occurs in a high strength matrix, this evolution saturates, and remarkable magnetic anisotropy is developed when Barkhausen noise in the transversal direction grows at the expense of the rolling direction. The contribution of residual stresses as well as the domain wall thickness is only minor, and the evolution of Barkhausen noise is driven by the density of the domain walls and their realignment.

## Introduction

Low alloyed steels (LAS) of low, medium, or high strength are frequently used for many applications in the automotive, civil (bridges), aerospace, or petrochemical industries^1,2^. Posing good machinability, hot formability, and weldability, these steels are very often proposed for the production of components due to the satisfactory ratio between their cost and functional properties. A variety of thermomechanical regimes in which these steels can be produced enable the customization of their matrix with respect to their fatigue resistance, resistance against friction and impact wear, fracture toughness, corrosion resistance, etc.^1^. LAS are deeply studied in order to better understand the complex mechanism of their deformation and to explore the contribution of some aspects that affect their functionality. Zhao et al.^3^ corrected the flow stress during hot forming to eliminate adiabatic heating and friction. Li et al.^4^ increased the strength of high-strength LAS by circular TiC particles. Yu et al.^5^ investigated the hardenability of high-strength LAS with respect to its crystallography and the corresponding hardness. Wang et al.^6^ studied the toughness of high strength LAS with respect to the Cu content. Alipooramirabad et al.^7^ studied the strain relaxation of welds in high strength LAS in situ by using neutron diffraction.

Monitoring components made of LAS after processing would be beneficial in order to reveal an unacceptable state of the microstructure or/and residual stress. Many conditions during the manufacturing process are kept constant, but some of them can fluctuate randomly or as a result of cutting tool wear, heterogeneity of delivered bodies, etc. For this reason, a fast and reliable technique employed for such a purpose could be helpful. LAS are ferromagnetic bodies containing a domain structure where neighboring domains are separated by domain walls (DWs). Due to the presence of pinning sites such as precipitates, grain boundaries, or dislocation tangles, the motion of DWs under a magnetic field altering in time is not smooth and occurs in the form of discontinuous and irreversible jumps^8,9^. Although each of the DWs in motion produces an electromagnetic pulse, the collective motion of the DWs occurs in the form of avalanches as a result of their clustering^10–12^. These overlapping pulses can be detected by a suitable coil on the free surface as magnetic Barkhausen noise (MBN)^9^.

LAS of variable strength have been already investigated by MBN. A previous article^13^ described the investigation in-situ as well as post-situ of the MBN in LAS with a yield strength (*σ*_*YS*_) of 235 MPa as a function of plastic straining and reported a significant magnetic anisotropy as well as attenuation of the MBN as a result of increasing dislocation density. Also, Schmidova et al.^14^ reported a remarkable magnetic anisotropy in interstitial free (IF) steels beyond the plastic instability. Antonio et al.^15^ showed that the grain and the corresponding domain structure fragmentation affected the MBN after plastic deformation. Piotrowski et al.^16^ measured the evolution of MBN after plastic deformation as a function of 90° and 180° DWs density. Kikuchi et al.^17^ found that MBN envelopes are shifted towards higher magnetic fields as a result of the cellular dislocation structure.

The remarkable increase in strength (*σ*_*YS*_ as well as ultimate – *σ*_*US*_) of LAS is, therefore, a result of the mechanical as well as the synergistic effect of thermal cycles. The *σ*_*YS*_ of LAS can exceed 1000 MPa. MBN is a function of the stress state^18,19^ as well as the microstructure (grain size, precipitate size and density, dislocation density, etc.^20–22^). The alteration of the steel matrix during hot rolling, therefore, affects the MBN emission as well. The systematic investigation with respect to MBN as a potential tool for LAS characterization is missing. Therefore, this study provides deep insight into the MBN emission of LAS of variable strength in which all important types of contributions to MBN are investigated.

## Experimental methods

Experiments were performed on LAS with nominal *σ*_*YS*_ values of, 355, 500, 700, 960, and 1100 MPa. LAS of nominal *σ*_*YS*_ 235 MPa represents the parental matrix whereas the LAS higher *σ*_*YS*_ are a product thermos-mechanical treatment of the parental steel. The LAS of variable strength were delivered in the form of 2000 mm × 1000 mm sheets (5 mm in thickness). The chemical composition of the parental steel can be found in Table 1.

**Table 1:** Chemical composition of investigated parental steels in wt%.

Yield strength (MPa)	Trademark	Fe	C	Mn	Si	P	S	Al
235	S235	bal	0.22	1.6	0.05	0.05	0.05	0.04

Samples of size 70 mm × 30 mm were cut for analysis. The preliminary investigation carried out by MBN as well as microhardness measurements revealed quite a remarkable shadowing effect of the surface layer of thickness of about 0.1 mm (remarkably thermally softened in contrast to the deeper regions). In order to investigate magnetic and other properties volumetric in nature (more or less the same within the thickness of the samples), the surface layer of thickness 0.15 mm was etched off (electrolytic process).

The different nominal *σ*_*YS*_ values of LAS are mostly a product of hot rolling conditions and the rate of cooling. The detailed conditions under which the delivered sheets were hot-rolled are not known, but these sheets represent the commercially available grades widely sold on the market. It is considered that the higher strength of LAS is due to higher energy consumed by the sheet during hot rolling as well as due to superimposing contribution of accelerated cooling rate. The true mechanical properties were investigated by the uniaxial tensile test on the samples of the dog-bone shape (overall length of 250 mm and width of 22.5 mm, the distance between shoulders of 50 mm, gauge length of 40 mm, gauge width of 14 mm, radius of 5 mm) using the device Instron 5985. True elastic strains were measured by the use of the Instron dynamic strain gauge extensometer 2620-602 on the length of 25 mm. Samples were investigated along the sheet rolling direction (RD) as well as the transversal direction (TD). The direction along the thickness of the sheets is referred to as ND. For each nominal *σ*_*YS*_ and the direction (RD or TD), three repetitive measurements were carried out.

**Figure 1:**
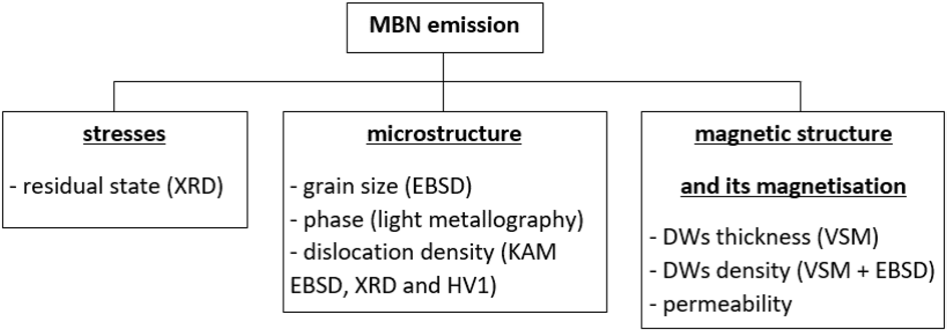
Brief list of aspects affecting MBN and experimental techniques employed for their analysis.

MBN in ferromagnetic bodies is a function of the stress state^18,19^, microstructure expressed in many terms^20–22^, as well as the alignment, thickness, and density of the DWs^22–25^. For this reason, true interpretation of MBN was carried out with respect to all the aforementioned aspects, see Fig. 1.

Electron backscatter diffraction (EBSD) analysis was used to explore the microstructure of the investigated samples. A scanning electron microscope ZEISS Auriga Compact equipped with the EDAX EBSD camera was used. The raw data were partially cleaned by one step of confidence index (CI) standardization and one step of grain dilatation. Only points with CI > 0.1 were used for the analysis. Only the areas separated by the high angle grain boundaries (misorientation > 15°) were recognized as grains. Note that the martensitic laths formed inside primary ferritic grains were considered in this study as separate grains because the phase boundary has the same effect on the motion of the DWs as the high angle grain boundary. The kernel average misorientaion (KAM) maps were calculated for the first neighbors only with the limit of 5°.

Hysteresis loops of the samples were measured using the vibrating sample magnetometer (VSM) Microsense EZ 9. For these measurements, we used cylindrical samples with a base diameter of about 4.2 mm and a height of about 2.5 mm. A maximal magnetic field of 1200 kA/m was applied in the base plane either along the RD or TD. The shapes of the measured curves were additionally modified with respect to the calculated demagnetizing factors^26^.

Initial magnetization curves (the dependence of the induction *B* on magnetic field *H* from the demagnetizing state) and reversible relative permeability at each point of the initial curve were measured by a modified DC fluxmeter-based hysteresis graph^27^ on ring-shaped samples (outer diameter of 24 mm, inner diameter of 18 mm, and height from 5 to 6 mm). The derivative of the initial magnetization curve in each point of the magnetization curve $\left[{H}_{0 }{,B}_{0}\right]$ determines the differential relative permeability ${\mu }_{dif}$

1 $${\mu }_{dif}=\frac{1}{{\mu }_{0}}{\left(\frac{dB}{dH}\right)}_{\left[{H}_{0 }{,B}_{0}\right]},$$

where ${\mu }_{0}$ is the permeability of vacuum.

The reversible relative permeability was measured using a lock-in amplifier reading of the induced voltage excited by a small AC magnetic field with the frequency of 10 Hz, causing exclusively reversible magnetic processes in the ferromagnet superimposed on the DC magnetic field *H*_0_. The reversible relative permeability is calculated using the equation

2 $${\mu }_{rev}=\frac{1}{{\mu }_{0}}\underset{\Delta H\to 0}{\mathrm{lim}}{\left(\frac{\Delta B}{\Delta H}\right)}_{\left[{H}_{0 ,}{B}_{0}\right]}.$$

The irreversible relative permeability ${\mu }_{irr}$ is consequently calculated as the difference between the differential and the reversible relative permeability

3 $${\mu }_{irr}={{\mu }_{dif}-\mu }_{rev}.$$

Residual stresses in the sheets (at the depth of 0.15 mm) were determined in the RD as well as the TD by the X-ray diffraction (XRD) technique (Proto iXRD Combo diffractometer, *Kα*_*1*_ and *Kα*_*2*_ of *{211}* planes, Cr*Kα*, Winholtz and Cohen method, *½s*_*2*_ = 5.75 TPa^−1^, s_1_ =  − 1.25 TPa^−1^). The microhardness HV1 was measured by the Innova Test 400TM device (1000 g load for 10 s, five repetitive measurements). In order to observe the microstructure of the matrix, the specimens of length 15 mm were cut along the RD, hot molded, ground, polished, and etched with 3% Nital.

Acquisition of the raw MBN signal was carried out by the RollScan 350 (mag. voltage of ± 5 V, mag. frequency of 125 Hz, sine profile, sensor S1-18–12-01). MBN was measured as the angular dependence with a step of 22.5° where the zero angle corresponds to the RD. The signals were filtered by the high pass filter (10 kHz) and low pass filter (1000 kHz) in the software package MicroScan 600. This software also extracts the conventional effective (rms) value of the Barkhausen noise, referred to as MBN. MBN envelopes were reconstructed on the basis of the filtered MBN signals, and the *PP* parameter as the position of the MBN envelope maximum in a magnetic field was analyzed as well. Finally, Micro Scan 600 also extracted information about the number of detected MBN pulses as well as the distribution function in which the number of MBN pulses was plotted as a function of their height. All MBN parameters were obtained by averaging ten consecutive bursts (five hysteresis cycles).

## Results of experiments and their discussion

### Mechanical properties

**Figure 2:**
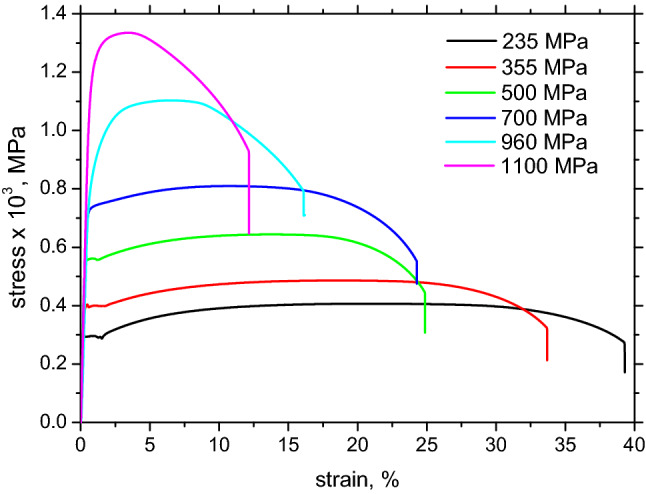
Engineering stress–strain curves, RD.

The mechanical properties provided in Table 2 were obtained from stress–strain curves depicted in Fig. 2. The true *σ*_*YS*_ is more as that the minimal guaranteed (the nominal one). The increase in *σ*_*YS*_ as well as *σ*_*US*_ (ultimate strength) is at the expense of the decrease in the elongation at break. The mechanism of *σ*_*YS*_ growth for LAS of nominal *σ*_*YS*_ values of 355 and 500 MPa is based mainly on grain refinement (discussed later). Therefore, one might expect that elongation at break could grow with *σ*_*YS*_ because this concept improves the strength as well as the toughness of the steel^1^.

**Table 2:** Mechanical properties obtained from stress–strain curves.

Nominal *σ*_*YS*_ (MPa)	True *σ*_*YS*_ (MPa)		True *σ*_*US*_ (MPa)		Elongation at break (%)		*σ*_*YS*_/*σ*_*US*_	
	RD	TD	RD	TD	RD	TD	RD	TD
235	312 ± 6	338 ± 3	406 ± 7	408 ± 3	39 ± 1.5	38 ± 0.4	0.77	0.82
355	408 ± 5	422 ± 11	485 ± 8	492 ± 9	34 ± 0.5	33 ± 0.4	0.84	0.87
500	572 ± 10	592 ± 14	643 ± 10	653 ± 7	25 ± 1.2	25 ± 1.6	0.87	0.89
700	764 ± 23	814 ± 10	829 ± 12	840 ± 6	24 ± 1.2	20 ± 1.2	0.94	0.96
960	987 ± 17	1058 ± 5	1101 ± 17	1113 ± 15	16 ± 0.7	14 ± 0.4	0.89	0.95
1100	1232 ± 15	1274 ± 6	1333 ± 15	1396 ± 12	12 ± 0.6	11 ± 1.2	0.92	0.91

However, this behavior was not found in this particular case. *σ*_*YS*_ and *σ*_*US*_ in the TD are greater than in the RD, and this evolution is reversed with respect to the elongation at break (see Table 2). This means that the work hardening mechanism based on dislocation multiplications and their mutual interaction is consumed earlier in the TD as a result of crystallographic heterogeneity developed during rolling. The growth of *σ*_*US*_ is lower than that of *σ*_*YS*_. For this reason, the ratio *σ*_*YS*_/*σ*_*US*_ increases gently with *σ*_*YS*_ but saturates early (see Table 2).

### Metallographic and EBSD observations

MBN is usually very sensitive to the microstructure; thus, the explanation of the significant aspects of the microstructure should be discussed in order to obtain a true interpretation of the MBN emission. More detailed information and deeper insight can be found in previous studies^1,28,29^. Mechanisms in which LAS can be strengthened are driven by grain refinement, phase transformation and the presence of precipitates^1,27^. Mechanical properties are given by their superimposing contribution as a result of the hot rolling temperature, energy consumed by the matrix, cooling rate, etc.^1,28,29^.

**Figure 3:**
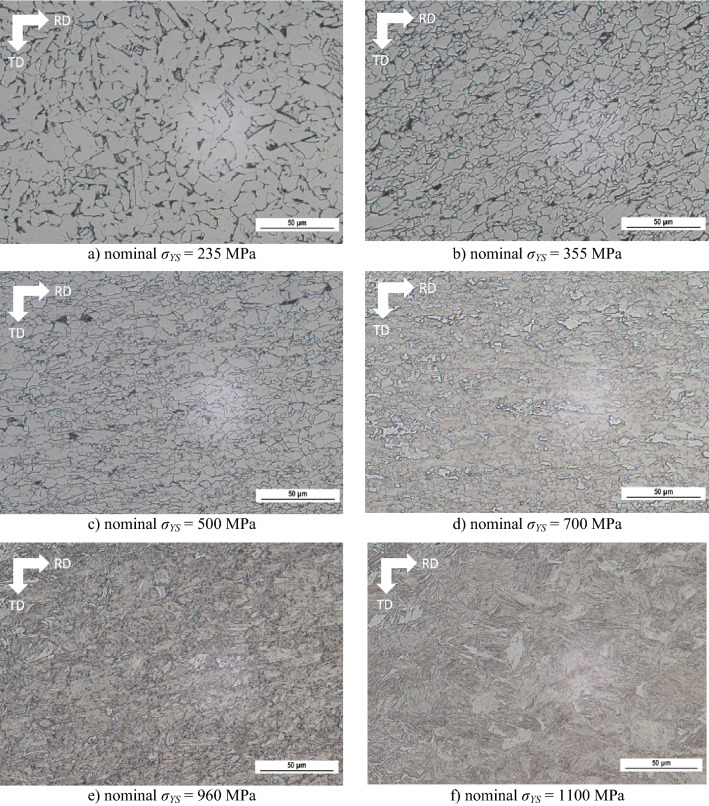
Metallographic images.

**Figure 4:**
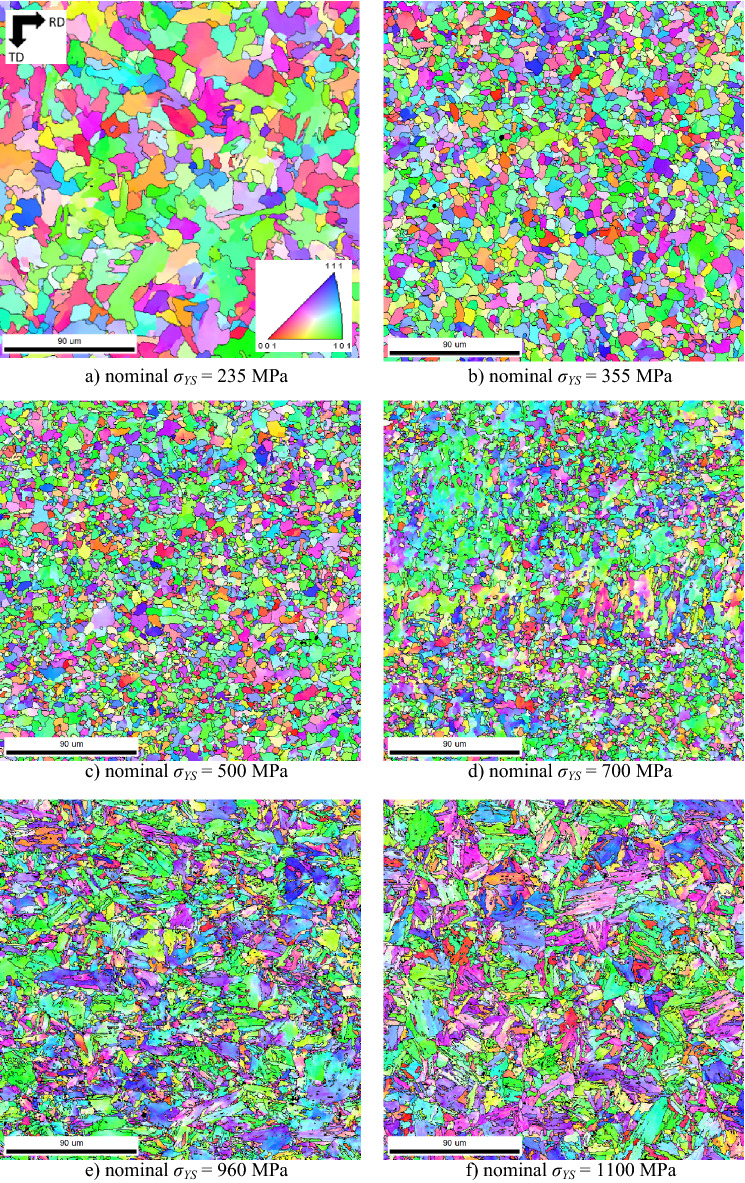
IPF EBSD figures.

Strengthening of LAS of *σ*_*YS*_ = 355 and 500 MPa is mainly based on grain refinement. The microstructure of LAS of *σ*_*YS*_ = 235, 355, and 500 MPa is fully ferritic and with localized pearlite islands, see Figs. 3 and 4. A fully ferritic matrix indicates lower cooling rates (air cooling considered) after hot rolling, and decreasing grain size with *σ*_*YS*_ indicates a higher austenite work hardening during hot rolling (higher density of suitable sites for ferrite nucleation).

**Figure 5:**
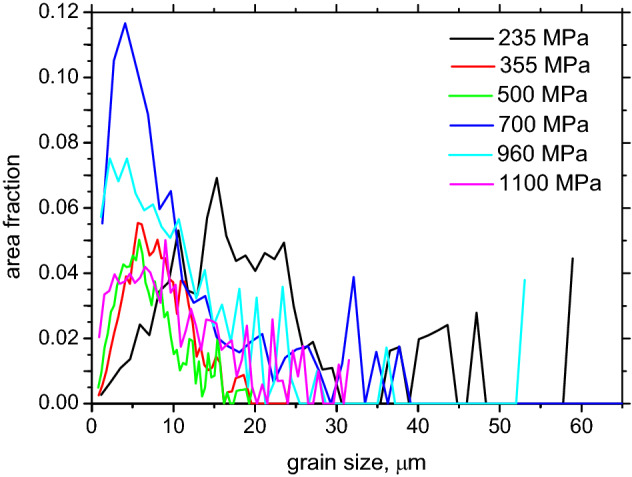
Distribution of grain size as a function of nominal *σ*_*YS*_.

The microstructure of LAS of *σ*_*YS*_ = 700 MPa is composed of ferrite + bainite as a result of an accelerated cooling rate^1,29^ (as compared with a fully ferritic matrix), see Figs. 3d and 4d. The higher *σ*_*YS*_ is due to the phase transformation and the corresponding retardation of dislocation. LAS of *σ*_*YS*_ = 960 MPa represent the complex matrix as a mixture of bainite + martensite (based on IQ EBSD images as reported in^28^), whereas LAS of *σ*_*YS*_ = 1100 MPa are fully martensitic. A progressive increase in *σ*_*YS*_ is given by the microstructure and corresponding mobility of dislocations as a result of increasing cooling rates^1^. These figures also show that more or less equiaxed ferrite grains are replaced by rough plate bainite and martensite. The distribution function of grain size *d* for all LAS is depicted in Fig. 5.

**Figure 6:**
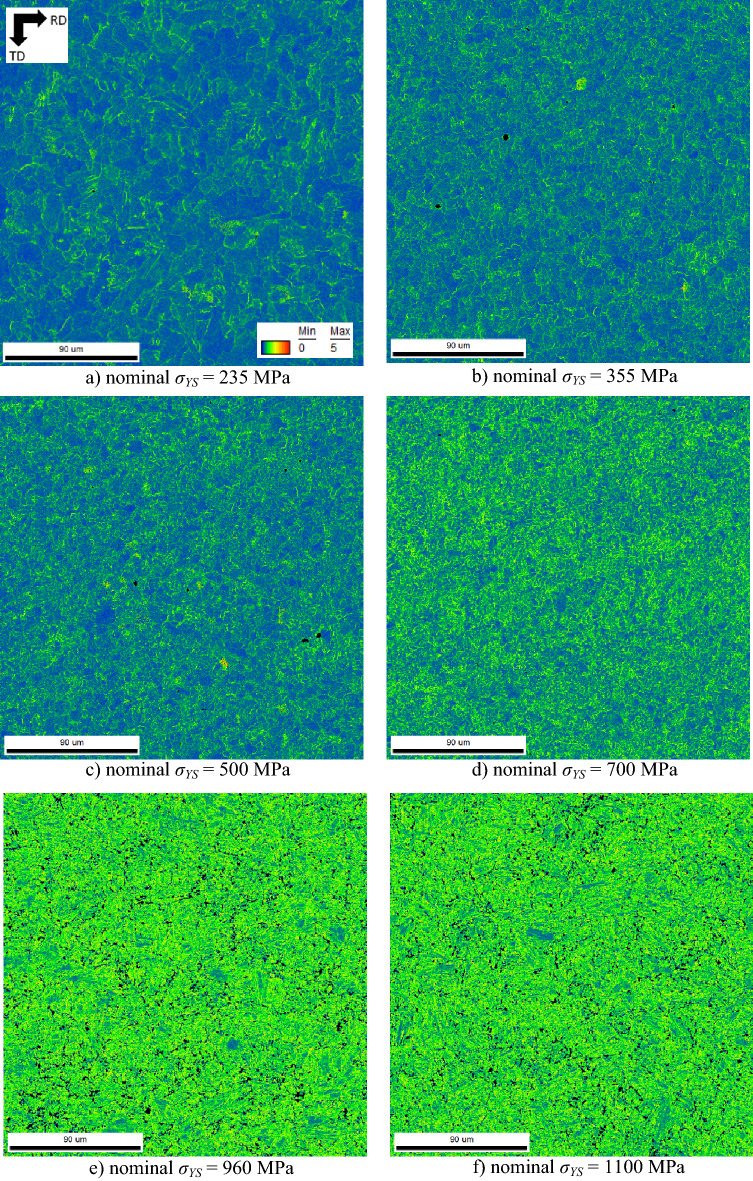
KAM maps, EBSD.

Dislocations in motion tend to be annihilated during hot rolling. However, the annihilation is incomplete and a certain fraction of dislocations is retained in the matrix and grows along with *σ*_*YS*_, see Fig. 6. Moreover, the phase transformation at an accelerated cooling rate avoids diffusion and promotes short distance shearing in the lattice. Figure 6, therefore, illustrates the increasing lattice misorientation attributed to the increasing dislocation density along with *σ*_*YS*_.

### Hardness and residual stresses

**Figure 7:**
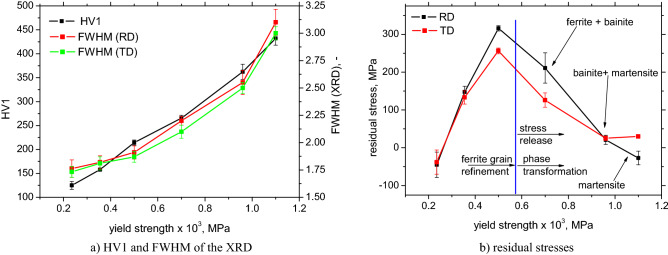
Evolution of HV1 and FWHM of the XRD and residual stresses as a function of the nominal *σ*_*YS*_.

Information about the increasing dislocation density also proves the HV1 measurement as well as the FWHM of the XRD ferrite patterns (FWHM of the XRD in these steels is mostly linked with the dislocation density^30^), see Fig. 7a. In particular, HV1 increases nearly linearly with *σ*_*YS*_. The ferrite grain refinement increases the amplitude of the tensile residual stresses, see Fig. 7b. As soon as the cooling rates are accelerated, the amplitude of the tensile stresses decreases because the phase transformation consumes a lot of energy stored in the matrix during hot rolling^1,28^. The evolution of the macroscopic residual stresses of I type (as depicted in Fig. 7b) in the RD and TD is very similar.

### DWs configuration and magnetization process

Information about the thickness, energy, as well as spacing of the DWs can be obtained when the constant of magneto-crystalline anisotropy *K*_1_ is measured from the hysteresis loops (by the use of the VSM technique) employing the Stoner-Wohlfarth model^31,32^:

4 $${H}_{A}=\frac{2{K}_{1}}{{\mu }_{0}{M}_{s}}=\frac{2{K}_{1}}{{J}_{s}},$$

where *H*_*A*_ is the anisotropy field and *M*_*s*_ is the saturation magnetization (obtained from the hysteresis loop as well). The 180° DWs thickness *δ* is driven by the minimum of the exchange and anisotropy energies^8,9,33^.

5 $$\delta = \, \pi \cdot {\text{sqrt}}\left( {|A|/K_{{1}} } \right),$$

where *A* is the exchange stiffness (1.26 × 10^–11^ J m^−1^ for Fe alloys^8,9^). The energy of the DWs* γ* is inversely proportional to the *δ*^8,30,31^.

6 $$\gamma = { 2}\pi .{\text{sqrt}}\left( {|A| \cdot K_{1} } \right).$$

Table 3 provides information about the measured *M*_*s*_, obtained *K*_1_, and the calculated *δ* and* γ*. It can be reported that the differences among the samples with respect to *M*_*s*_, *K*_1_, *δ*, and* γ* are only minor, whereas the measured coercivity *H*_*c*_ gradually increases along with *σ*_*YS*_ as a result of the increasing density of lattice imperfection (especially the dislocation density, see Figs. 6 and 7a).

**Table 3:** Magnetic parameters obtained from the VSM measurements.

Nominal yield strength, MPa	*M*_*s*_, kA m^−1^	*H*_*c*_, kA m^−1^	*K*_1_, J m^−3^	*δ*, nm	*γ*, 10^3^ J m^−2^
235	1563	0.45	1.0 10^5^	35	7.0
355	1539	0.52	9.6 10^4^	36	6.9
500	1580	0.65	9.8 10^4^	36	7.0
700	1563	0.67	8.4 10^4^	38	6.5
960	1520	0.82	9.4 10^4^	36	6.8
1100	1526	1.44	9.8 10^4^	35	7.0

Having information about *M*_*s*_, *γ*, and the grain size *d* distribution from the EBSD observations (see Fig. 5), the spacing *D* of the DWs can also be calculated^33^:

7 $$D= \sqrt{\frac{18\gamma {\mu }_{0}\frac{d}{2}}{{M}_{s}^{2}}}$$

**Figure 8:**
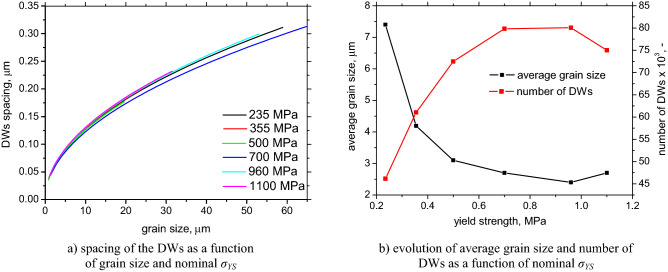
Evolution of spacing of the DWs, average grain size, and number of DWs (per 0.0625 mm^2^).

The spacing *D* of the DWs can be calculated within the grains of the different sizes *d* as well as the average spacing of the DWs and the corresponding number of DWs per investigated area, see Fig. 8. Figure 8a clearly demonstrates that *D* is mainly a function of the grain size and the evolution of *D* versus *d* is nearly the same for all LAS as a result of their similar *M*_*s*_ (see Table 3). However, the decreasing *d* along with *σ*_*YS*_ increases the density of the DWs and a corresponding number of DWs, see Fig. 8b.

**Figure 9:**
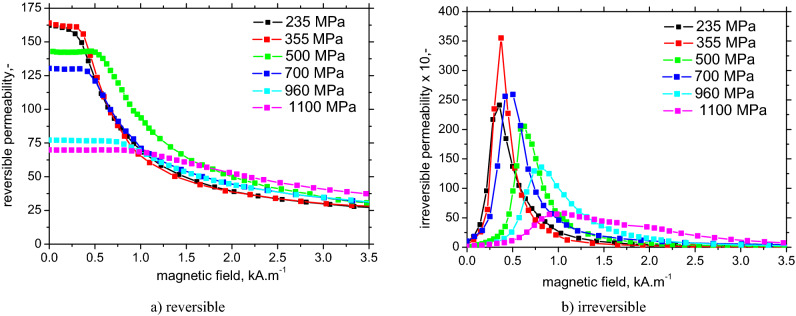
Dependence of reversible and irreversible permeability vs. magnetic field with nominal yield strength *σ*_*YS*_ as a parameter.

The tendency of the maximum value of the real part of the complex permeability (at a low magnetic field) to decrease with an increase in the yield strength* σ*_*YS*_ is clearly visible in Fig. 9a. It can be assumed that the higher value of *σ*_*YS*_ is then the higher value of the density of dislocations, acting as obstacles in the displacement of domain walls, occurring in the materials and the real part of the complex permeability decreases. When the maximum value of the real part of the complex permeability is higher, then the tendency to decrease with the magnetic field occurs at a lower magnetic field (except for the sample with *σ*_*YS*_ = 700 MPa) because most reversal magnetization processes were realized at lower magnetic fields. The irreversible component (Fig. 9b) tends to decrease with *σ*_*YS*_, the maximum is shifted towards the higher magnetic fields, but this evolution is not straightforward. The evolution of the permeabilities is mainly driven by the increasing density of pinning sites (especially the dislocation cells) with increasing *σ*_*YS*_. These sites increase the magnetic field necessary to unpin DWs and the corresponding domains and make the free path of the irreversible DWs in motion shorter. Moreover, it also makes the free distance between the neighboring pinning sites shorter for reversible processes such as the bending or/and reversible rotation of DWs (promote the irreversible motion at the expense of the reversible one).

### Barkhausen noise measurements

**Figure 10:**
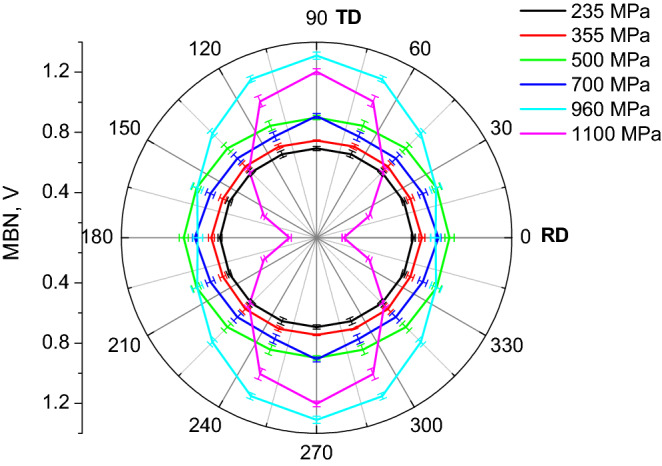
Angular distribution of MBN as a function of nominal *σ*_*YS*_.

**Figure 11:**
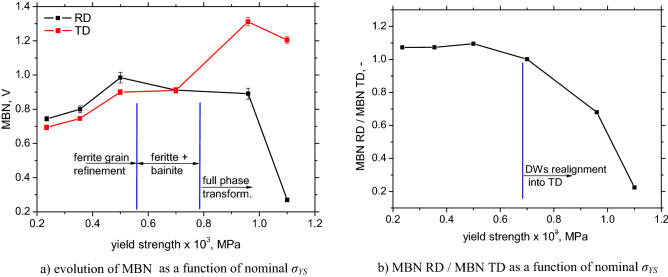
Evolution of MBN and MBN anisotropy.

Figures 10 and 11a demonstrate that MBN grows with ferrite grain refinement (for LAS of *σ*_*YS*_ = 235, 355, and 500 MPa) as a result of increasing density of DWs and the corresponding irreversible jumps of DWs contributing to MBN, as was discussed earlier by Sakamoto et al.^23^ or Anglada-Rivera et al.^24^. Sakamoto et al.^23^ also reported that the effective value of the MBN signal is inversely proportional to the square root of the grain size:

8 $${\text{MBN }}\left( {{\text{rms}}} \right) \, = {\text{ C}}_{{\text{g}}} {\text{d}}^{{ - {1}/{2}}}$$

when *C*_*g*_ is a grain size dependent constant. Magnetic anisotropy expressed in terms of MBN in the RD and TD is quite low for the ferritic LAS when MBN is only slightly more in the RD compared to the TD, see Figs. 10 and 11. As soon as the phase transformation takes place, this ratio is reversed and the aforementioned anisotropy becomes valuable for LAS of *σ*_*YS*_ = 960 and 1100 MPa, see also Figs. 10 and 11. The TD becomes an easy axis of magnetization, whereas the RD is a difficult one. The degree of this magnetic anisotropy progressively increases with *σ*_*YS*_, see Fig. 11b. The growth of MBN in the RD at the expense of the TD is linked with the realignment of the DWs into the TD (discussed later).

MBN can also be expressed in terms of a number of detected MBN pulses *n* and their height *X*_*i*_ as follows:

9 $$MBN (rms)=\sqrt{\frac{1}{n}\sum_{i=1}^{n}{X}_{i}^{2}}$$

**Figure 12:**
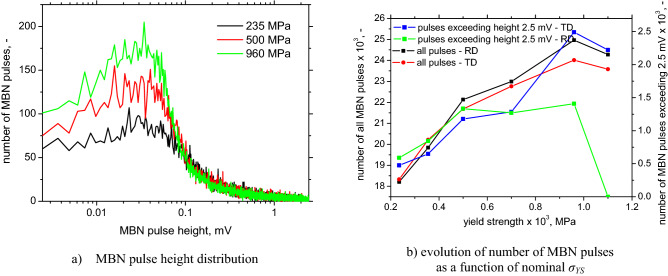
MBN pulse height distribution and evolution of number of MBN pulses.

The distribution function of MBN pulses depicted in Fig. 12a shows an increasing number of weak pulses for LAS of higher *σ*_*YS*_. Figure 12b also demonstrates remarkable differences among the samples with respect to the number of strong MBN pulses exceeding 2.5 mV. Comparing Figs. 11a and 12b, a strong correlation between MBN and the number of especially strong MBN pulses is observed.

**Figure 13:**
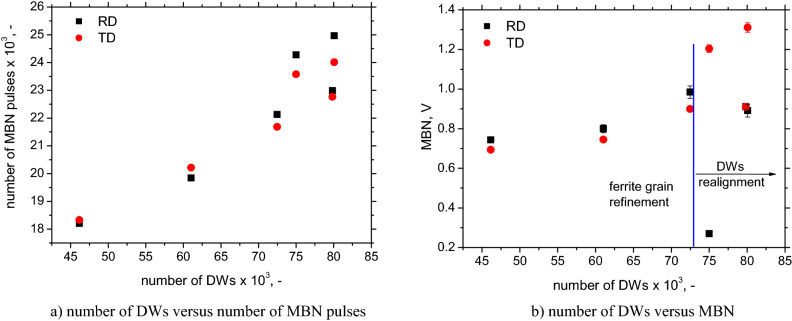
Number of DWs versus number of MBN pulses and number of DWs versus MBN.

The number of all detected MBN pulses for the RD and TD is quite similar, especially for LAS of lower *σ*_*YS*_, see Fig. 12b. For this reason, MBN is driven by two major factors; (i) the number of detected pulses; (ii) the alignment of DWs. The first one dominates in a fully ferritic matrix; the second one prevails when the phase transformation takes place, see Fig. 13b. Figure 13a depicts a strong correlation between the number of detected MBN pulses and the number of calculated DWs. However, the number of calculated DWs is much higher than that detected by the sensing coil despite the fact that the sensing coil area is much larger (about 4 mm^2^) than the EBDS observed area (only 0.0625 mm^2^). This remarkable controversy is driven by the clustering of DWs^10–12^ when the motion of the DWs is a collective process in the form of avalanches. Being so, electromagnetic pulses originating from the sole DWs overlap in time. Moreover, the limited sampling frequency of 6.7 MHz for the data acquisition also plays a certain role.

**Figure 14:**
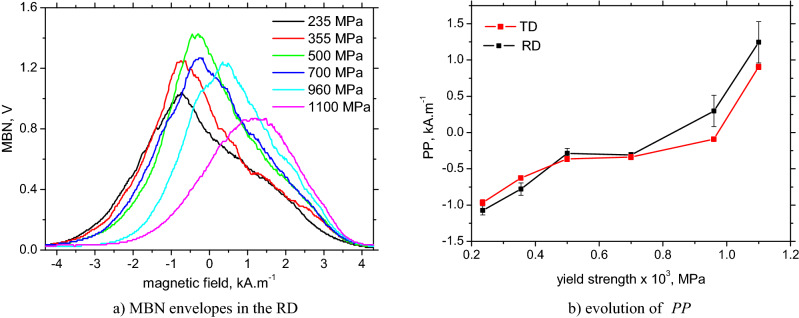
MBN envelopes in the RD and evolution of *PP*.

**Figure 15:**
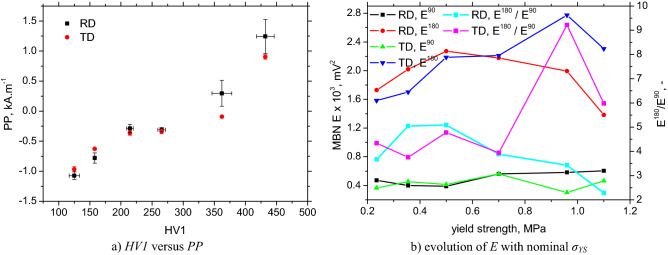
Number of DWs versus MBN; evolution of *E* with nominal *σ*_*YS*_.

Increasing *HV1*, *H*_*c*_ as a result of increasing dislocation density also affects the MBN envelope positions and the corresponding *PP*, see Fig. 14. MBN is shifted towards the higher magnetic field along with *σ*_*YS*_ due to increasing opposition of pinning sites (higher density of pinning sites). The *PP* for LAS of lower *σ*_*YS*_ is similar, but the realignment of DWs into the TD for LAS of *σ*_*YS*_ = 960 and 1100 MPa makes the *PP* in the TD slightly lower compared with the RD, see Fig. 14b. This is a result of the initial phase of the rotation of DWs during magnetization in the RD, whereas this phase is strongly attenuated when magnetization is in the TD^22,34^. Figure 15b clearly proves that the *PP* is a function of the matrix hardness and the corresponding density of lattice imperfection, as reported earlier^13^.

The sensitivity of the *PP* parameter for LAS characterization is very good as a result of the straightforward correlation of *PP* with *HV1* or/and *H*_*c*_^13,20,25^. Expressed in other words, *PP* can be easily and directly linked with *σ*_*YS*_ because *σ*_*YS*_ strongly correlates with *HV1*. On the other hand, increasing the pinning strength of the matrix plays only a minor role in the effective value of the Barkhausen noise signal because MBN grows with *σ*_*YS*_. Applied magnetic fields are strong enough to unpin DWs for all LAS. The intensity of the field used to produce the maximum avalanche can be directly linked with the position of the MBN envelope maximum in a magnetic field which is expressed in *PP*, see Fig. 14. On the one hand, increasing the density of the pinning site reduces the free path of the motion of DWs. On the other hand, increasing the frequency of events when DWs in motion encounter a pinning site contributes to the higher number of pulses produced by the electromagnetic pulses.

Certain contributions of residual stresses to MBN can be considered for ferritic LAS only when MBN increases along with the growing amplitude of tensile stresses, see Figs. 7b and 11a. As soon as the phase transformation takes place, the influence of micro stresses prevails and macroscopic residual stresses play no role^8,20,21^. The correlation between irreversible permeability and MBN is weak (compare Figs. 9a and 11a). Irreversible permeability decreases with *σ*_*YS*_ as contrasted against MBN (especially in the TD). It should be noted that their physical origin is quite different. Permeability is linked more with the rate of sample magnetization as a result of domain realignment, whereas MBN is mainly associated with the irreversible motion of DWs^9^. Although the motion of domains and their surrounding DWs is mutually interconnected, the magnetization rate of a body composed of larger domains could be higher, but MBN can be weaker due to the lower density of DWs (as is demonstrated in this particular case).

The realignment of DWs in the TD that was observed in this study has been already proved and reported earlier in Trip and S235 steels after the tensile test^13,35^. In particular, the behavior observed in the study employing S235^13^ can be closely linked with the similar behavior in this study because S235 can be considered as the parental body (despite a little bit of altered chemistry) for all LAS. The evolution of MBN in the RD and TD is very similar despite the different regimes of exerted load (cold uniaxial tension in^13^ and multiaxial with superimposing thermal cycle employed in this study).

The realignment of DWs into the TD and the different mechanisms of the motion of DWs when the sample is magnetized in the TD and RD can also be proved by employing the simplified model of Martinez-Ortiz et al.^36^ for the calculation of MBN energies based on MBN envelopes. The authors proposed a model in which the MBN energy associated with pure 180° DWs irreversible motion *E*^*180*^ can be calculated from the MBN envelope in the region near the main peak, whereas the rotation or/and 90° motion of DWs can be detected especially in the initial phases of an envelope beyond the first detected pulse (*E*^*90*^). Figure 15b depicts similar *E*^*180*^ and *E*^*90*^ as well as lower *E*^*180*^/*E*^*90*^ for ferritic LAS in the RD as well as the TD. On the other hand, *E*^*180*^ in the TD grows at the expense of lower *E*^*90*^, which results in a high *E*^*180*^/*E*^*90*^, whereas *E*^*180*^ decreases in the RD together with *E*^*180*^/*E*^*90*^. This behavior indicates the initial rotation of DWs in the RD, as explained earlier^34^.

Finally, it should be noted that MBN in LAS martensite is significantly higher than that originating from, for example, bearing steels^21^. Although very fine needle martensite with a high density of DWs can be expected in bearing steels, the high C content as well as the additional alloying elements produces a matrix with a much higher dislocation density and especially a high density of precipitates (especially Fe_3_C) that strongly hinder DWs in motion (and/or makes the free path of DWs in the irreversible motion very short), which makes the MBN approximately one order lower^20,21^.

## Conclusions

The main findings can be summarized as follows:

- MBN in LAS of the ferritic structure is mostly driven by grain refinement and the density of DWs,
- the phase transformation as a result of accelerated cooling rates produces the matrix with a remarkable magnetic anisotropy when MBN in the TD is more than that in the RD,
- accelerated cooling rates tend to align the DWs into the TD at the expense of the RD,
- the number of detected MBN pulses strongly affects MBN and correlates with *d* and the density of DWs,
- *PP* can be easily employed for LAS characterization of variable *σ*_*SY*_ and the corresponding *HV1*,
- the contribution of residual stresses and fine precipitates to MBN is minor.

## Data Availability

The raw data required to reproduce these findings cannot be shared easily due to technical limitations (some files are too large). However, authors can share the data on any individual request (please contact the corresponding author by the use of the mailing address).

## References

[1] Vervynckt S, Verbeken K, Lopez B, Jonas JJ (2012). Modern HSLA steels and role of non-recrystallisation temperature. Int. Mater. Rew..

[2] Fonstein, N. *Advanced High Strength Sheet Steels*. first ed., Springer International publishing, Switzerland, 2015. 10.1007/978-3-319-19165-2.

[3] Zhao M, Huang L, Li Ch, Li J, Li P (2021). Evaluation of the deformation behaviors and hot workability of a high-strength low-alloy steel. Mater. Sci. Eng. A.

[4] Li S, Yu H, Lu Y, Lu J, Wang W, Yang S (2021). Effects of titanium content on the impact wear properties of high-strength low-alloy steels. Wear.

[5] Yu YS (2021). New insight into the hardenability of high strength low alloy steel from the perspective of crystallography. Mater. Let..

[6] Wang J, Wang S, Xi X, Wang G, Chen L (2021). The role of copper in microstructure and toughness of intercritically reheated coarse grained heat affected zone in a high strength low alloy steel. Mater. Char..

[7] Alipooramirabad H, Paradowska A, Reid M, Ghomashchi R (2022). Effect of holding time on strain relaxation in high-strength low-alloy steel welds: An in-situ neutron diffraction approach. J. Manuf. Proc..

[8] Cullity BD, Graham CD (2009). Introduction to the Magnetic Materials.

[9] Jiles D (2016). Introduction to Magnetism and Magnetic Materials.

[10] Bohn F (2018). Playing with universality classes of Barkhausen avalanches. Sci. Rep..

[11] Hansen UB (2022). Magnetic Bloch oscillations and domain wall dynamics in a near-Ising ferromagnetic chain. Nat. Commun..

[12] Tadić B, Mijatović S, Janićević S, Spasojević D, Rodgers GJ (2019). The critical Barkhausen avalanches in thin random-field ferromagnets with an open boundary. Sci. Rep..

[13] Neslušan M, Jurkovič M, Kalina T, Pitoňák M, Zgútová K (2020). Monitoring of S235 steel over-stressing by the use of Barkhausen noise technique. Eng. Fail. Anal..

[14] Schmidová E (2022). Monitoring of plastic straining degree of components made of interstitial free steel after uniaxial tensile test by the use of Barkhausen noise technique. Steel Res. Int..

[15] Antonio PP, Campos MF, Dias FMS, Campos MA, Capo-Sanchez J, Padovese LR (2014). Sharp increase of hysteresis area due to small plastic deformation studied with magnetic Barkhausen noise. IEEE Trans. Mag..

[16] Piotrowski L, Augustyniak B, Chmielewski M, Hristoforou EV, Kosma K (2010). Evaluation of Barkhausen noise and magnetoacoustic emission signals properties for plastically deformed Armco iron. IEEE Trans. Mag..

[17] Kikuchi H, Ara K, Kamada Y, Kobayashi S (2009). Effect of microstructure changes on Barkhausen noise properties and hysteresis loop in cold rolled low carbon steel. IEEE Trans. Mag..

[18] Liu J, Tian GY, Gao B, Zeng K, Zheng Y, Chen J (2020). Micro-macro characteristics between domain wall motion and magnetic Barkhausen noise under tensile stress. J. Magn. Magn. Mater..

[19] Sorsa A, Santa-aho S, Wartiainen J, Souminen L, Vippola M, Leviskä K (2018). Effect of shot peening parameters to residual stress profiles and Barkhausen noise. J. Nondestruct. Eval..

[20] Neslušan M, Minárik P, Čilliková M, Kolařík K, Rubešová K (2019). Barkhausen noise emission in tool steel X210Cr12 after semi-solid processing. Mater. Char..

[21] Neslušan M, Čížek J, Kolařík K, Minárik P, Čilliková M, Melikhová O (2017). Monitoring of grinding burn via Barkhausen noise emission in case-hardened steel in large-bearing production. J. Mater. Process. Technol..

[22] Neslušan M, Trojan K, Haušild P, Minárik P, Mičietová A, Čapek J (2020). Monitoring of components made of duplex steel after turning as a function of flank wear by the use of Barkhausen noise emission. Mater. Char..

[23] Sakamoto H, Okada M, Homma M (1987). Theoretical analysis of Barkhausen noise in carbon steels. IEEE Trans. Magn..

[24] Anglada-Rivera J, Padovese LR, Capó-Sanchez J (2001). Magnetic Barkhausen noise and hysteresis loop in commercial carbon steel: Influence of applied tensile stress and grain size. J. Magn. Magn. Mater..

[25] Neslušan M (2021). Barkhausen noise emission in Fe-resin soft magnetic composites. J. Magn. Magn. Mater..

[26] Osborn JA (1945). Demagnetizing factors of the general ellipsoid. Phys. Rev..

[27] Birčáková Z, Kollár P, Weidenfeller B, Füzer J, Fáberová M, Bureš R (2015). Reversible and irreversible DC magnetization processes in the frame of magnetic, thermal and electrical properties of Fe-based composite materials. J. Alloys. Comp..

[28] De Ardo AJ, Hua MJ, Cho KG, Garcia GI (2009). On strength of micro alloyed steels: an interpretive review. Mater. Sci. Technol..

[29] Zhang HK, Xiao H, Fang XW, Zhang Q, Logé RE, Huang K (2020). A critical assessment of experimental investigation of dynamic recrystallization of metallic materials. Mater. Des..

[30] Mittemeijer EJ, Scardi P (2004). Diffraction analysis of the microstructure of materials.

[31] Pop NCO, Caltun F (2011). Jiles-atherton magnetic hysteresis parameters identification. Acta Phys. Pol. A.

[32] Jiles DC, Atherton DL (1986). Theory of ferromagnetic hysteresis. J. Mag. Magn. Mater..

[33] Chikazumi S (2005). Physics of ferromagnetism.

[34] Neslušan M (2019). Barkhausen noise emission in hard-milled surfaces. Mater..

[35] Neslušan M (2022). Measurement of the rate of transformation induced plasticity in TRIP steel by the use of Barkhausen noise emission as a function of plastic straining. ISA Trans..

[36] Martínez-Ortiz P, Pérez-Benitez JA, Espina-Hernández JH, Caleyo F, Hallen JM (2015). On the estimation of the magnetic easy axis in pipeline steels using magnetic Barkhausen noise. J. Magn. Magn. Mater..

